# Pre-screening of endomysial microvessel density by fast random forest image processing machine learning algorithm accelerates recognition of a modified vascular network in idiopathic inflammatory myopathies

**DOI:** 10.1186/s13000-025-01608-3

**Published:** 2025-01-31

**Authors:** Alessandro Massaro, Gerardo Cazzato, Giuseppe Ingravallo, Nadia Casatta, Carmelo Lupo, Angelo Vacca, Florenzo Iannone, Francesco Girolamo

**Affiliations:** 1 Department of Engineering, LUM University “Giuseppe Degennaro”, Casamassima, Italy; 2 Section of Molecular Pathology, Department of Precision and Regenerative Medicine and Ionian Area (DiMePRe-J), University of Bari “Aldo Moro”, Bari, 70124 Italy; 3 Diapath SpA, Martinengo, Italy; 4 Engineering and Applied Science Department, University of Bergamo, Bergamo, Italy; 5 Guido Baccelli Unit of Internal Medicine, Department of Precision and Regenerative Medicine and Jonian Area-(DiMePRe-J), University of Bari Aldo Moro, Bari, Italy; 6 Section of Rheumathology, Department of Precision Regenerative Medicine and Ionian Area (DiMePRe-J), University of Bari Aldo Moro, Bari, Italy; 7 Unit of Human Anatomy and Histology, Department of Translational Biomedicine and Neuroscience “DiBraiN”, University of Bari, Bari, Italy

**Keywords:** FRF image processing, Myositis, Neovascularization, Necrotizing myopathy, CD31

## Abstract

Biomarkers for discrimination among different subgroups of idiopathic inflammatory myopathies (IIM) are difficult to identify and may involve multiple laboratory tests and time-consuming procedures. We assessed the potential for artificial intelligence (AI) to extract features such as density of endomysial microvessels based on automatic analysis of the CD31^+^ vascular network on muscle biopsy images. We also assessed the potential of this technique to save time and its agreement rate with analyses based on the manual selection of microvessels from the same images. A total of 84 images from 84 patients with IIM, diagnosed between 2014 and 2020, were retrieved and analyzed using the Fast Random Forest (FRF) technique. We built a lightweight and explainable algorithm for calculating the pixel percentage of CD31^+^ endomysial capillaries. The FRF technique applied on images of CD31-stained muscle sections achieved a good performance in the recognition of microvessels by estimating their density over a standard area corresponding to a sample of microscope image. The time spent for this analysis was 90% less than the manual choice of microvessels (estimated time considering the computational time and the time spent to manually detecting the microvessels features). The good performance of the FRF demonstrates that the CD31 pixel percentage of endomysial capillaries is sufficient for a correct estimation. Finally, the paper proposes a procedure to integrate AI in the pre-screening process.

**Supplementary Information:**

The online version contains supplementary material available at 10.1186/s13000-025-01608-3.

## Introduction

Idiopathic inflammatory myopathies (IIM) are a heterogeneous group of autoimmune muscle diseases characterized by progressive muscle weakness, extracellular release of creatine kinase as a sign of sarcolemmal disintegration after exercise, chronic endomysial inflammation and vascular network alterations. IIM can be classified into several subgroups: dermatomyositis (DM; including amyopathic dermatomyositis), antisynthetase syndrome (ASS), immune-mediated necrotizing myopathy (IMNM), sporadic inclusion body myositis (sIBM), polymyositis (PM) and overlap myositis (OM) on the basis of clinical, serological features (including the presence/absence of different myositis-specific auto-antibodies), and the specific histopathology [1]. A systematic assessment of the vascular network in different IIMs by IHC staining with the endothelial marker CD31 revealed a prominent endomysial capillary density (CD) in anti-HMGCR^+^ IMNM, whereas a reduced CD was calculated in PM, ASS, and OM [2].

Confirming the increased capillary density in anti-HMGCR^+^ IMNM patients using automated calculation methods can be useful for discriminating IMNM from PM patients, that both have similar clinical and laboratory parameters but a significantly different CD [2]. The easy, rapid recognition of changes in the endomysial vascular network could serve not only as a useful biomarker to better classify IIM patients into more specific groups, but also have beneficial implications on diagnosis, prognosis, and treatment [3]. In addition, advanced imaging techniques and artificial intelligence (AI) can help in identifying specific regions of modified vascular networks, where the molecular pathways involved can be investigated, leading to a better understanding of the specific pathogenesis and the identification of potential therapeutic targets [4].

Traditional methods of image acquisition and analysis based on manual choice of microvessels as multiple regions of interest can be time-consuming procedures. However, recent advances in acquisition technology and the development of efficient algorithms for image analyses are continually reducing the time required for these procedures [5]. Considering the recent surge in the use of AI to support clinical and diagnostic procedures [6], automatic image analysis techniques can discover specific image features during the training process of the deep learning method, allowing the saving of time as compared to traditional methods. In the present paper, to enhance the image analysis speed in quantitative imaging, we have employed a possible advanced technique to extract image features, namely the Fast Random Forest (FRF). The FRF technique is a re-implementation of the Random Forest classifier (RF) operating in the Weka framework and features a good performance in computational cost and memory allocation. The FRF was initially applied in industrial applications involving image processing [7] and, successively, in medical imaging to detect, among other features, anomalous cell aspects in Malignant Melanoma [8] and in screening for Naevoid Melanoma in dermatopathology [9], to segment 3D images [10] and to model object shapes [11]. Specifically, in the proposed work, the FRF algorithm is adopted to identify microvessels in the whole slide images (WSI) of immunolabeled muscle sections. The algorithm classifies clusters of pixels embedding the shape of the CD31^+^ vessels, providing output as the probabilistic map indicating the probability of identifying these shapes into a specified image region [12]. Furthermore, as the FRF a supervised algorithm, it allows the creation of a classifier model (named training model) detecting the same classes (features) in other new testing images [13]. The possibility of selecting the images for the classifier allows optimization of the targeting of the features to be extracted, avoiding redundancies and wrong information about images containing artefacts [14, 15]. Indeed, the FRF also allows effective segmentation under challenging acquisition conditions, such as the presence of artefacts. The FRF technique has been successfully used to calculate the CD31^+^ endomysial pixel percentage in muscle biopsies obtained from a single center cohort of IIM patients.

## Materials and methods

### Muscle tissue

The muscle sections for CD31 immunostaining derived from single open biopsies of muscle tissue obtained for diagnostic purposes from 84 patients with clinically and pathologically confirmed IIMs already described in a previous published paper [2]. The analysis was performed prior to start any treatment on muscle biopsies from 8 patients with anti-HMGCR^+^ IMNM (2 patients exposed and 6 patients not exposed to statins), 5 patients with anti-SRP^+^ IMNM, 8 patients with seronegative IMNM (no MSA nor myositis-associated autoantibodies). Additional muscle samples were taken from 21 patients with adult DM, 8 patients with PM, 8 patients with systemic sclerosis-OM (SSc-OM), 8 patients with ASS (6 Jo1^+^ and 2 PL7^+^), 5 patients with PM with mitochondrial pathology (PM-Mito), 3 patients with adult mitochondrial myopathy (MM), 10 patients with sIBM, and 6 age-matched healthy subjects, used as controls. The 6 normal control specimens were derived from apparently healthy subjects with mildly elevated serum levels of CK but without any histological abnormality, including the absence of inflammatory infiltrates or MHC class I up-regulation. The specific clinical-pathological diagnoses of IMNM, DM, PM, OM, sIBM, PM-Mito, and ASS myositis were made according to the defined published criteria [16–20]. All procedures performed in this study were in accordance with the ethical standards of the 1964 Helsinki Declaration and its later amendments. The study was reviewed and approved by the Medical Ethics Committee of the Regional Policlinico University Hospital of Bari (study n. 6229, approval n. 84762, 2020/11/06; comitatoetico@policlinico.ba.it). All patients gave signed informed consent to the diagnostic and research analyses and specimen inclusion in a muscle biobank. The acquired images were obtained with an Olympus Vanox-T light microscope (Olympus, Hamburg, Germany) equipped with a high-resolution video camera (SPOT Insight; Diagnostic Instruments, MI, USA) and the whole-slide morphometric analysis scanning platform Aperio Scanscope CS (Leica Biosystems, Nussloch, Germany). All the slides were scanned at the maximum available magnification (40×).

### Overall FRF framework

The FRF model is applied to detect anomalous pixels highlighting the modified vascular network. Specifically, the millimeter raw images (microscopic images) are first converted into a gray scale images, and successively, the training model to be used to automate the recognition of the anomalies of the testing images is constructed. The training process is performed by selecting, using ellipses, the classes to be recognized. The classes are clusters of pixels representing a combination of gray scaled pixels arranged into a matrix enclosed into the ellipses. Three main labelled target classes are defined: the three classes are enough to recognize the background, isolated elements (endomysial microvessels) and agglomerates (perimysial vessels). For each class, different regions (ellipses) are selected in the same images, thus optimizing the training model. The testing was performed on twelve new images not used for the training process and considered suitable for the analysis (see also supporting materials). The FRF engine allows the features to be extracted as combinations of clusters of pixels, targeting the specific class, and provides, as output a probabilistic image indicating the higher probability of finding regions matching with the specific classes. Finally, the platform estimates the percentage of area of pixels with the greter probability. More details are discussed in the next section, listing all the steps followed.

### FRF model and platform functionalities

**Fig. 1:**
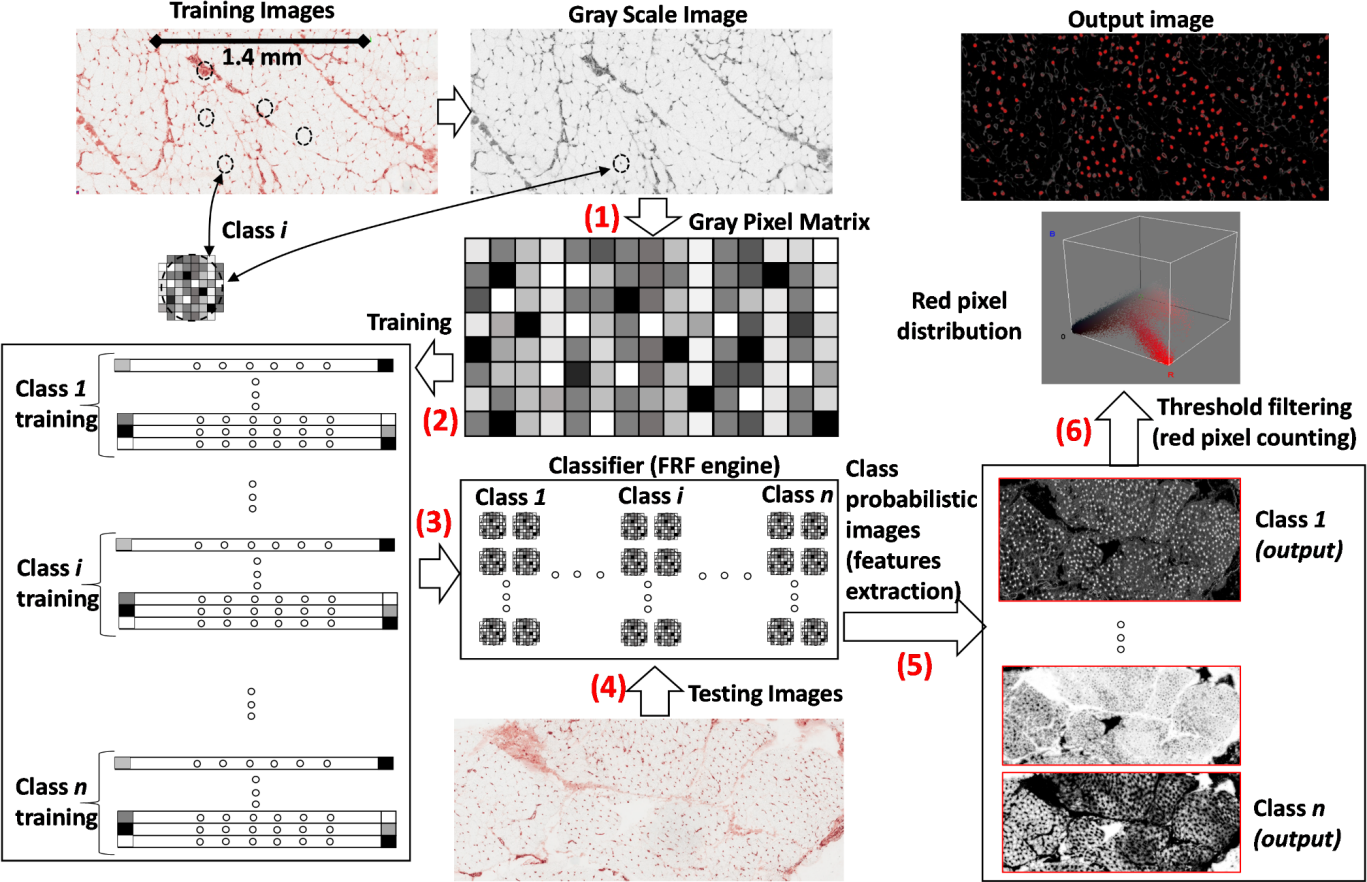
FRF model detecting anomalous pixels. The workflow is characterized by different phases indicated by numbers from 1 to 6: (1) conversion to gray scale of the original raw image (on a millimeter scale), obtaining a full gray pixel image; (2) training process of each image by selecting the classes by surrounding ellipses (clusters of pixels defining the classes 1, 2 and 3 as combinations of pixels with a different intensity); (3) construction of the FRF training model using the three labelled target classes of the supervised FRF algorithm; (4) testing of new unclassified images processed by the FRF engine; (5) features extraction (output images of the platform) as probabilistic images (as regard the new images, a probabilistic image is extracted for each class, indicating the higher probability of finding regions matching with the specific classes); (6) filtering (output images of the platform) of the pixels with a greater probability of obtaining the spatial distribution of the red pixels that will be automatically counted to estimate the risk level, shown as the red pixel percentage indicator (the threshold setting is the same as in the simple counting manual procedure so as to match the two approaches)

**Fig. 2:**
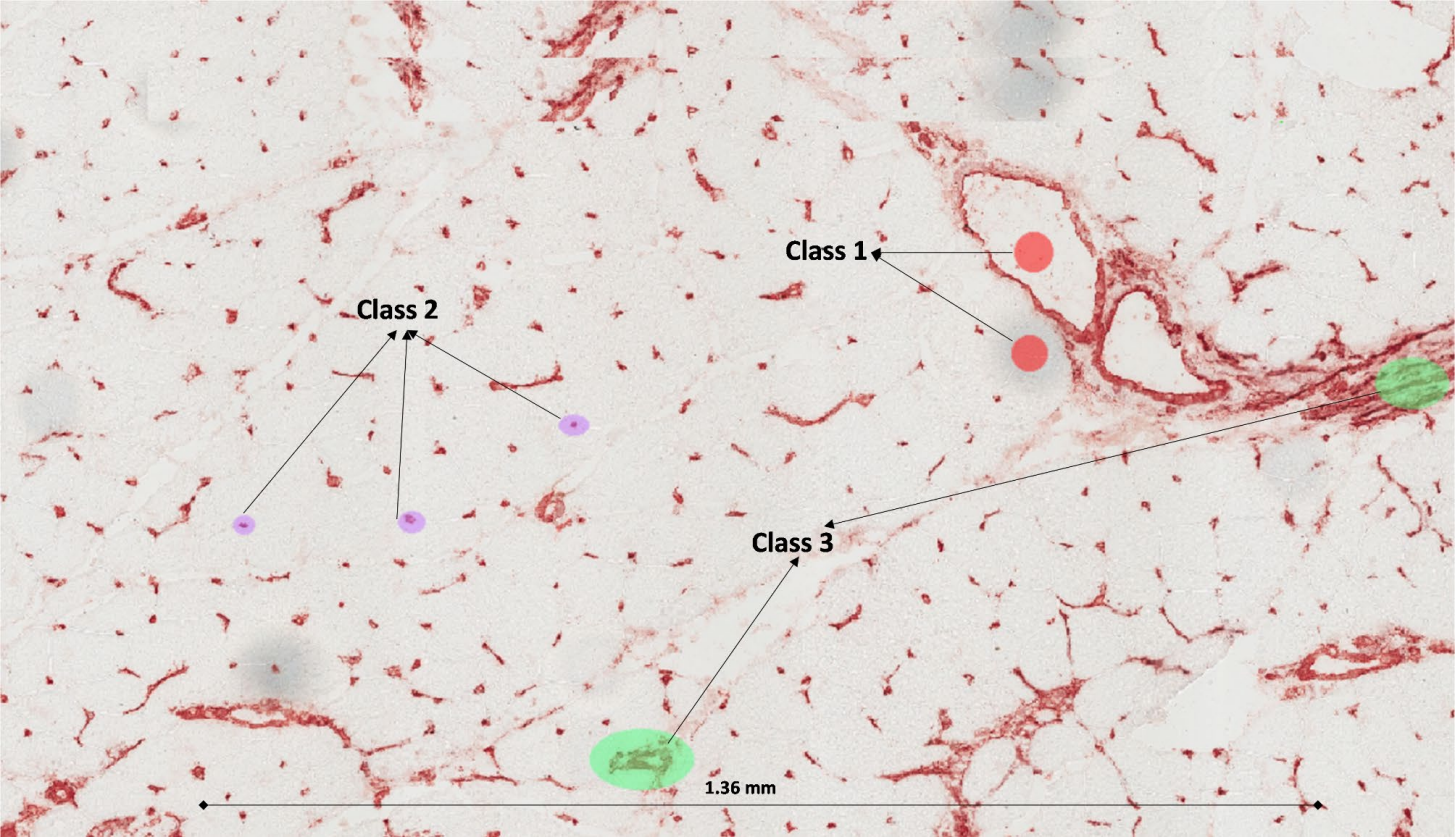
Example of the supervised approach defining three training classes using the FRF platform. The red ellipses demonstrate class 1 (empty background); the purple ellipses demonstrate class 2 (isolated elements = endomysial microvessels); the green ellipses demonstrate class 3 (aggregate elements = perimysial large vessels)

The FRF model adopted for image-processing to detect CD31^+^ endomysial microvessels is illustrated in Fig. 1, and follows six different stages:

- Stage 1 (selection of the training images): 84 initial images acquired with the two different systems at different known resolutions were selected for a second step selection of the training process (these differences allow the error gap in the training stages to be reduced);
- Stage 2 (gray scale conversion): all the selected images are automatically converted into gray scale images (gray pixel matrix containing all the image features except colors);
- Stage 3 (training process): for each final selected image suitable for the training, three main classes are identified to train the overall FRF classifier of the image processing platform (class 1: white spaces devoid of information, class 2: isolated endomysial microvessels, and class 3: perimysial vessels). To support the reproducibility of the proposed approach, we illustrate in Fig. 2 an example of a training process performed on a single image; the final classifier is constructed by adding the information on classes of other images. The possibility of selecting the same class on the same image allows the number of images needed for the training process to be significantly reduced.
- Stage 4 (testing images): the testing images are loaded and processed in the platform, obtaining the classification of the three classes as clusters of pixels contained in the images. The tested images are not used for the training process (data unseen by the training model), avoiding the overfitting and properly assessing the model’s performance.
- Stage 5 (probabilistic images): in the whole image for each class, the probabilistic maps are generated as topographic maps with pixels tending towards white color, indicating a higher probability;
- Stage 6 (threshold filtering and pixel counting): the pixels with an intensity gray value between 45 and 255 are filtered for each selected probabilistic image; these filtered pixels were enhanced by imposing a red color (output image of the selected class), counting their distribution, reported as the percentages of threshold pixels in the whole image.

The threshold setting of the step 6 is the same as in the simple counting manual procedure, so as to compare the two approaches.

The platform also provides the following algorithm performance parameters: Precision, Recall, FMeasure, Receiver Operating Characteristic Curve (ROC), Fallout, and Threshold. The *Precision* estimates the accuracy of positive predictions, the *Recall* measures the completeness of positive predictions, the *ROC* curve estimates the sensitivity as a function of the false positive rate (True Positive Rate versus False positive Rate), the Area Under Curve (AUC) is the total area estimated under the ROC curve, the *Fallaout* indicates the false positive rate, and the *Threshold* function evaluates the performance of a classification model by measuring the degree of separation between the positive and negative distributions (the model is better at separating the positive from negative cases when *T* = 1).

1 $$\:Precision=\frac{True\:Positive}{True\:Positive+False\:Positive}$$

2 $$\:Recall=\frac{True\:Positive}{True\:Positive+False\:Negative}$$

3 $$\:FMeasure = 2\frac{{Precision \cdot \:\:Recall}}{{Precision + Recall}}$$

4 $$\:Fallout=\frac{False\:Positive}{False\:Positive+True\:Negative}$$

5 $$Threshold = Recall - False\,Positive\,Rate$$

The FRF platform integrates the free open source Weka java-based libraries.

### The business process modelling and notation (BPMN) pre-screening protocol

The BPMN is an international standard graphical notation [21]- [22] (ISO/IEC 19510:2013) suitable to formulate a new protocol in medicine [7, 23] and in general to map healthcare processes [24]. In the proposed paper, the BPMN is adopted to formulate the pre-screening protocol Endomysial Microvessels integrating the supervised FRF algorithm as an engine supporting the discrimination of suspect cases based on a specific threshold of endomysial microvessel density.

### Statistical analysis

Comparison between-the two methods of measuring endomysial vessel density was performed using simple linear regression analysis with GraphPad Prism (release 6.0, GraphPad Software, La Jolla, USA). Differences were considered statistically significant at a *p*-value of < 0.001.

## FRF results

To measure the density of microvascular network in human muscle diagnostic biopsies and to construct the training model for the FRF classifier, three main classes of pixels have been defined: class 1 qualifies the white spaces, class 2, the isolated endomysial microvessels, and class 3, the perimysial large vessels. In Fig. 2 an example of identification of the three classes on the same image is illustrated: only three small pixel areas are enough to identify the classes for FRF algorithm processing. The proposed platform allows the areas enclosed by ellipses to be identified during the classification process: these areas are clusters of pixels defining the classes 1, 2 or 3 as combination of pixels with a different intensity, defining the features to be recognized. This identification is not required for a stable constructed training model. Only 12 images are already enough to construct a good algorithm training process. For each image, the areas containing appropriate pixels are manually enclosed by ellipses (see Fig. 2), defining the main three classes indicating a white background (class 1), isolated endothelial cells of endomysial microvessels (class 2), and perimysial large vessels (class 3). The FRF training classifier does not improve in performance by increasing the images for classification beyond 12. The 12 images chosen for the classification consider different microscope detecting conditions (light, adjustment, etc.) to compensate for any possible calculus error during the classification of the testing process (Step 4).

**Fig. 3:**
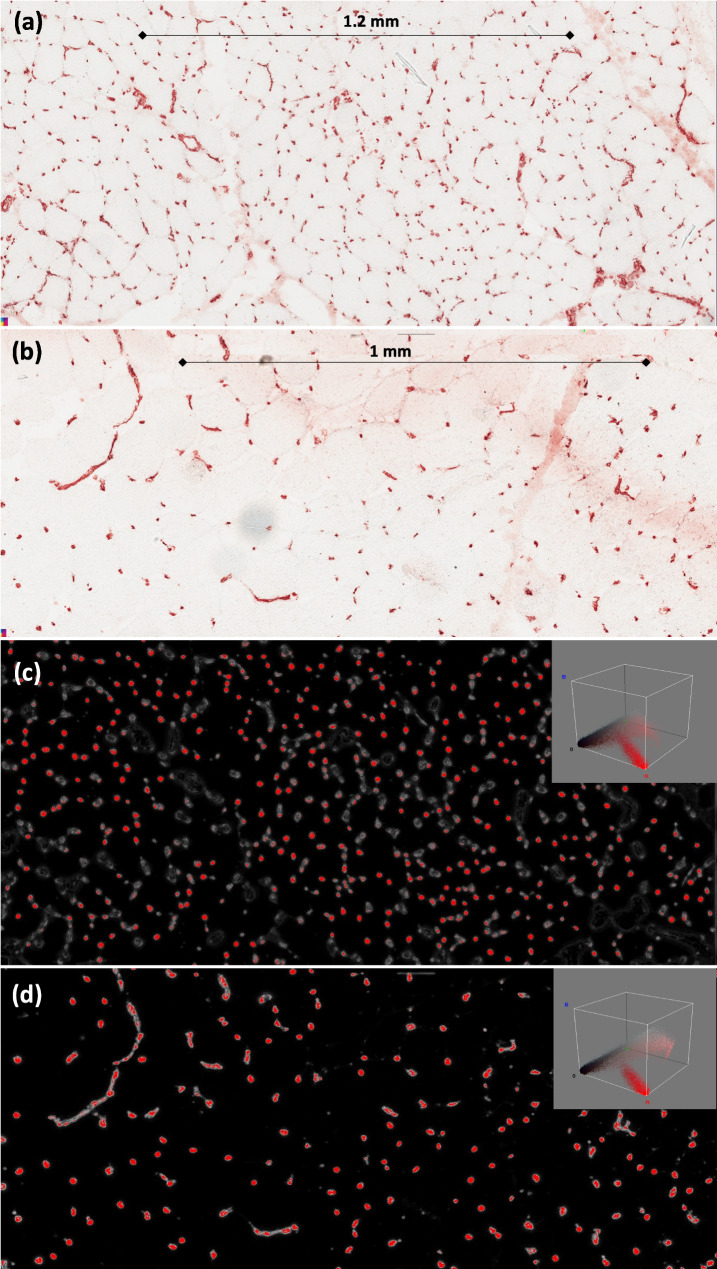
Representative images of CD31-immunolabeled human muscle tissue showing the CD31^+^ vascular network in a female anti-HMGCR^+^ IMNM patient (**a**) and a female PM patient (**b**), (**c**, **d**) FRF processed images highlighting the quantifiable red pixels of endomysial microvessels, class 2 for the FRF classifier. The red pixel distribution is shown in the insets. The pixel distribution is related to the isolated endothelial cells of class 2, the black region contains other parts of the muscle tissue not included in the class 2. These excluded regions could contain agglomerates of blood vessels (the red color indicates a high probability of finding isolated endothelial cells only). As mentioned in the main text, the different density of vascular network is useful to discriminate between the various kind of IIM

**Fig. 4:**
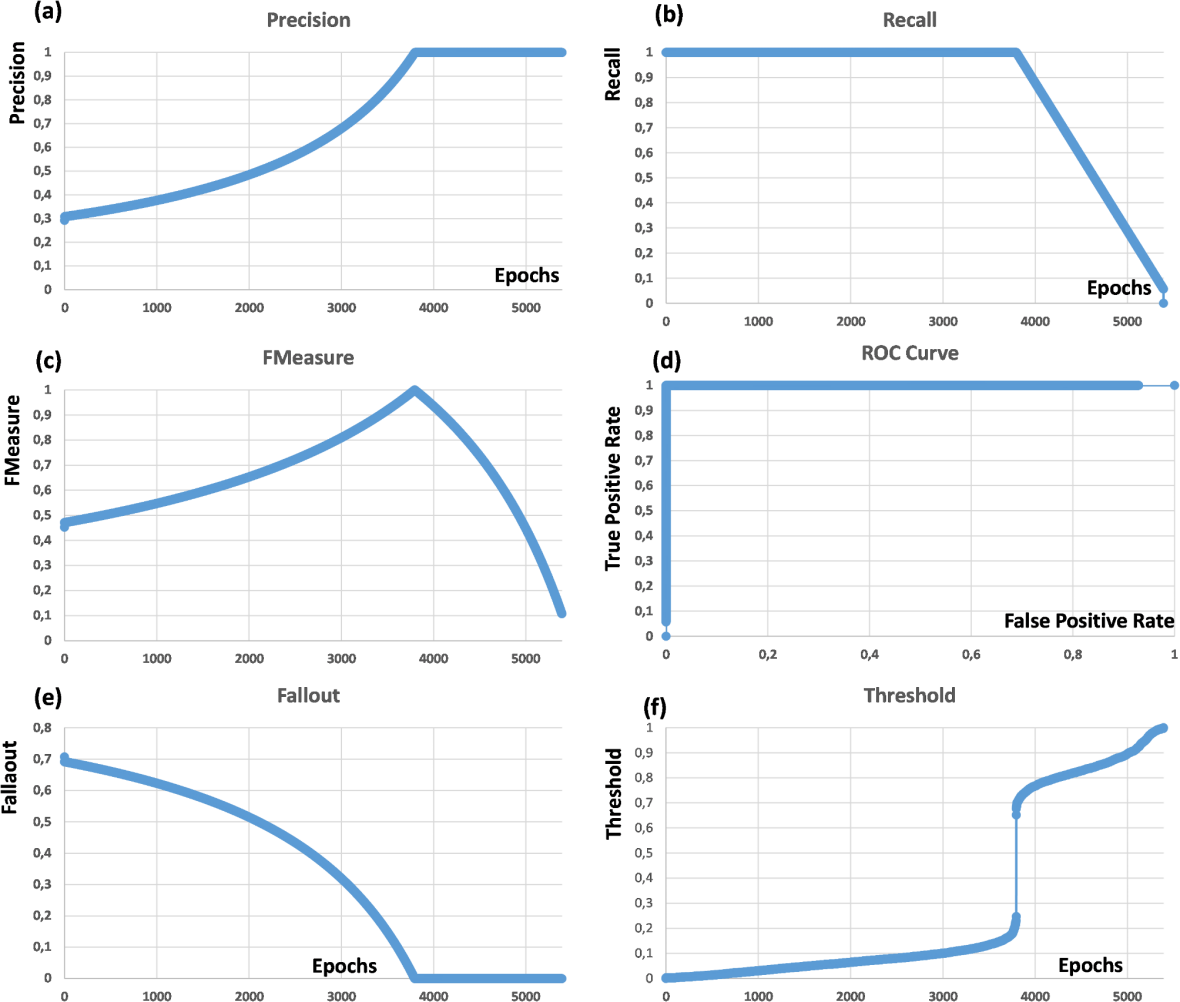
FRF Performance parameters: (**a**) *Precision* versus epochs; (**b**) *Recall* versus epochs; (**c**) *FMeasure* versus epochs; (**d**) ROC curve estimating the sensitivity as a function of the false positive rate (True Positive Rate versus False positive Rate); (**e**) *Fallout* versus epochs; (**f**) *Threshold* versus epochs. After increasing the training images from 3 to 12, the epochs sufficient to acheive the convergence did not change significantly

**Fig. 5:**
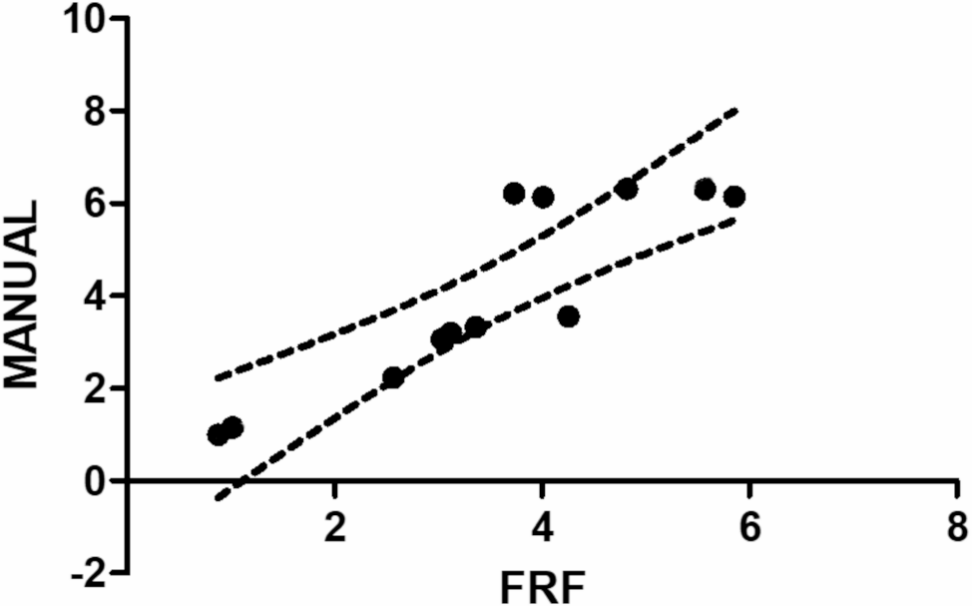
Linear regression graph of comparison between FRF and manual methods for counting endomysial microvessels

FRF image processing was performed for the 84 photomicrographs derived from 84 patients with IIM. A representative example of the obtained results is shown in Fig. 3. The FRF probabilistic images of the CD31 endomysial microvascular network provide the quantifiable pixels of microvessel density serving for further accurate analyses and comparison (Fig. 3c, d). The precision parameter (probability of algorithm error) has estimated the algorithm performance. A *Precision* of 1 is achieved after about 5390 iteration steps (epochs) executed by the FRF (Fig. 4). Few seconds are necessary to reach 5390 iterations using a standard PC (11th Gen Intel(R) Core(TM) i5-1135G7, 2.42 GHz). The *Recall* parameter of the chosen FRF processing analysis algorithm also showed an excellent performance (Fig. 5). Similar *Precision* and *Recall* trends were found only for the 12 images obtained using the Aperio Scanscope, an automatic scanning platform, whereas the other 72 images derived from a traditional microscope and video camera obtained poorer performances due to the smaller acquired areas.

**Fig. 6:**
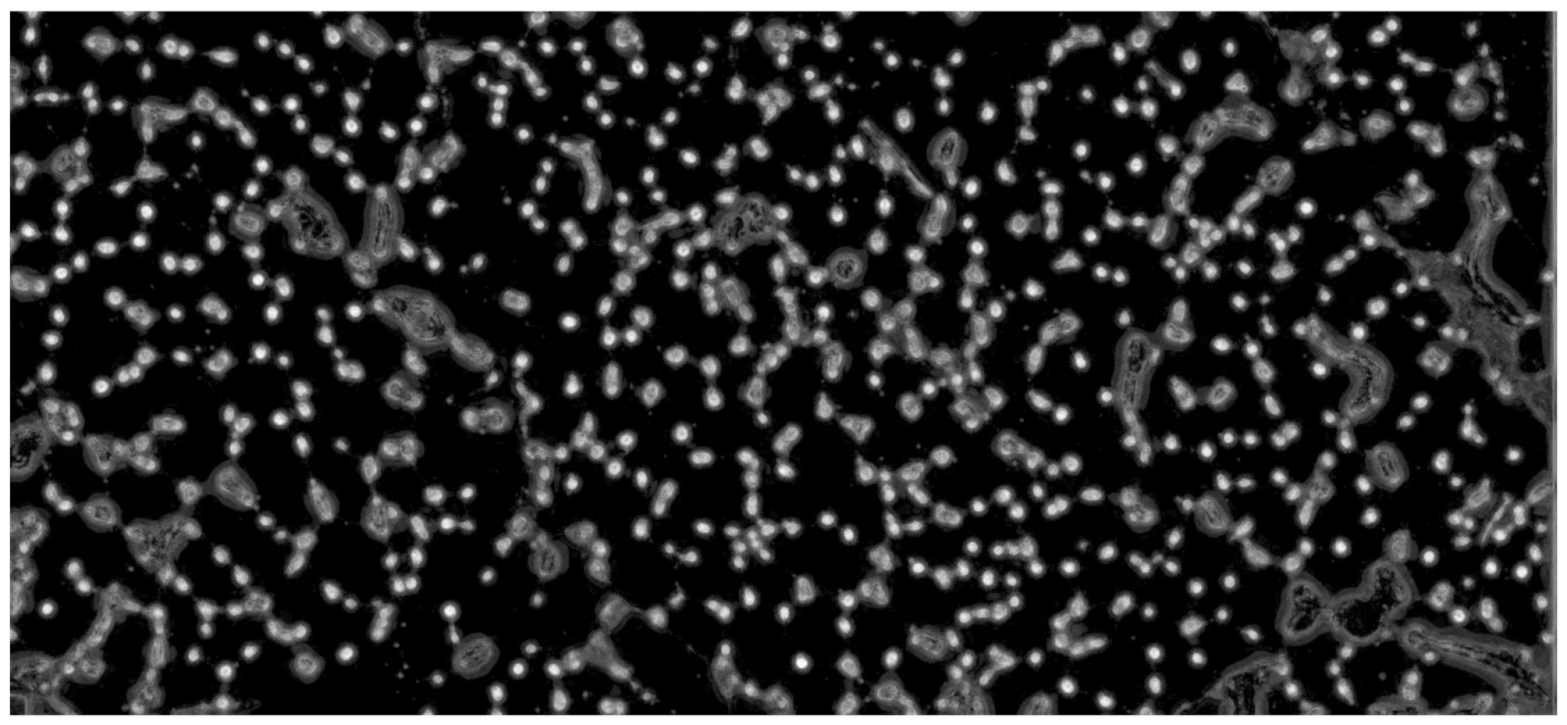
Probabilistic map of class 2 identifier of image shown in Fig. 3(a)

In order to show how the platform extracts the highest probability, the probabilistic map of the image processed in Fig. 3(c) is shown in Fig. 6: only the pixels with the highest intensity (near the white area indicating the highest probability) will be highlighted in red. The map of Fig. 6 is obtained by fixing the same threshold as for the other analyzed images.

Other experimental checks proved that the FRF algorithm performance trend did not change after increasing the training images. For each testing images, the performance parameters were estimated as very similar to those presented in the Fig. 4, highlighting the systematic calculus approach of the FRF model. As expected *Precision* and *Recall* show an inverse relationship (best performance achieved at 5390 epochs), the *FMeasure* is quite stable over a large probability range, confirming the *Precision* and *Recall* trends, the *ROC* curve highlights the ideality of the FRF classifier, the *Falloout* indicates a good decrease of the false positive rate, and the *Threshold* shows a clear separation between the positive and negative distributions. The very good performance results demonstrate that the FRF algorithm is a good alternative to the Convolutional Neural Network (CNN) applied in biomedical image processing (both the techniques exhibit a very close performance [25]). In Table 1 shows a comparison between different techniques Artificial Neural Network (ANN), CNN, Random Forest (RF), and FRF. The FRF algorithm proposed in this study is optimized for cell image processing [8, 9].

**Table 1:** Comparison of AI image processing performance (Area under curve –AUC–)

ANN [25]	RF [25]	CNN [25]	FRF (proposed study)
0.861	0.988	0.991	≅1

The simple linear regression analysis between FRF algorithm measures and manual counting of endomysial vessels shows a sufficient correlation coefficient (r^2^: 0.7919; F:38.05; p: 0.0001; Fig. 5).

All the images are processed by using a Core i5 2.4 75 GHz/16 GB RAM processor. The complexity of the model is decreased thanks to the possibility of selecting the same class in different regions (cluster of pixels enclosing a class) of a single image. By means of this approach, only about 20 s. are necessary for training on a single image (the computational time is a function of the number of ellipses representing the classes). The total computational cost of the training is the sum of the times necessary for the training of each image. About 30 s. are enough for the testing process and for the plotting of the probabilistic images. The main limitation of the FRF image processing lies in the variable image brightness (raw images having different degree of brightness) requiring a possible preliminary setting to obtain the same pixel intensities as in other images which could be acquired by another microscope. Furthermore, very dark images may lead to algorithm failure, incorrect training and incorrect probability estimation. For these reasons, a pre-selection of images is necessary.

## Pre-screening procedure

**Fig. 7:**
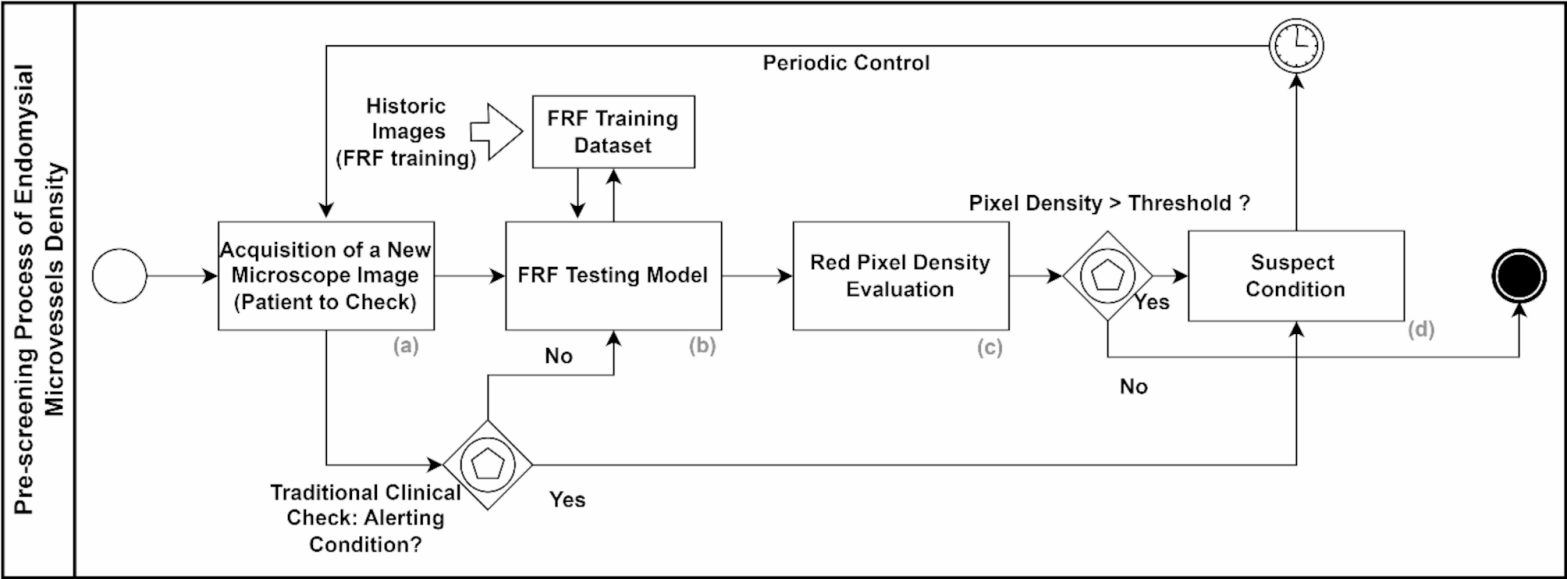
BPMN workflow modelling the pre-screening process of endomysial microvessel density alerting condition by means of microscope image analysis and integrating FRF processing. A microscopic image of the biopsy is taken and checked by a pathologist for any urgent pathology such as acute inflammation. If none is found, the image of tissue immunostained for CD31 is analyzed for capillaries and stratified according to the result

The proposed FRF model is a useful tool to support clinical investigations for pre-screening of anomalous endomysial microvessels density. In Fig. 7, the procedure to integrate FRF analysis into a pre-screening protocol merging traditional analyses with FRF is illustrated. Specifically, the BPMN workflow defines the procedure to define a suspect condition by through image analysis and processing. The BPMN approach is suitable for inclusion in workflow protocols in medicine [21] and for pre-screening processes [9]. The proposed protocol is characterized by the following steps:

- The process starts with the correct acquisition of the microscope image of the patient to be checked: the images must be detected if possible using the same microscope and using the same image setting to decrease the risk of FRF algorithm error (resolution, contrast, saturation, luminosity, etc.);
- The FRF testing is performed by selecting on the image the class to be checked for (in the proposed process, class 2 is observed); the same image is adopted to improve the training FRF model;
- The platform estimates the red pixel distribution in the analyzed image, indicating the endomysial microvessels density;
- The decision is made to repeat the examination when the FRF algorithm estimates a density of class 2 pixels exceeding a threshold based on analyses of images derived from unaffected individuals.

The procedure highlights the point that the patient should be not periodically biopsied when both the traditional and the FRF check show no suspect conditions. The threshold is placed in a % area change. For the cases analyzed in our cohort of IIM patients, a threshold greater than 4% can refer to patients requiring further attention for their muscle angiogenic potential and confidently assigned an IMNM rather than a PM diagnosis.

## Conclusion

The proposed FRF model has been adopted to support the clinical investigation for an IIM diagnosis. Specifically, the method is suitable for the pre-screening of patients by estimating their endomysial microvessels density. The FRF algorithm automates a supplementary check of patients, adopting image processing. The high performance FRF method allows the precise computed analysis of sub-optimal images because the prior machine training is able to mediate the calculation error. Furthermore, few images are already enough to construct a good training model. The paper demonstrates that FRF could be a good alternative to traditional pre-screening procedures, suggesting a new clinical process that can define conditions suggestive of IMNM if a higher endomysial microvessel density is calculated. This method allows more than 90% time saving during capillary density estimation. The very good performance of the FRF algorithm, indicated by the *Precision* parameter equals to 1 after different epochs, shows that there is no need to compare other deep learning algorithms in order to improve the performance.

## Electronic supplementary material

Below is the link to the electronic supplementary material.

Supplementary Material 1

## Data Availability

No datasets were generated or analysed during the current study.
