# Supplementary material for: Pre-screening of endomysial microvessel density by fast random forest image processing machine learning algorithm accelerates recognition of a modified vascular network in idiopathic inflammatory myopathies
Source: Diagn Pathol. 2025 Jan 31;20:13. doi: 10.1186/s13000-025-01608-3 (PMC11783852; doi:10.1186/s13000-025-01608-3)
Supplement: Supplementary file 1 — Supplementary Material 1 [file 13000_2025_1608_MOESM1_ESM.pdf]

Photo : **1326**

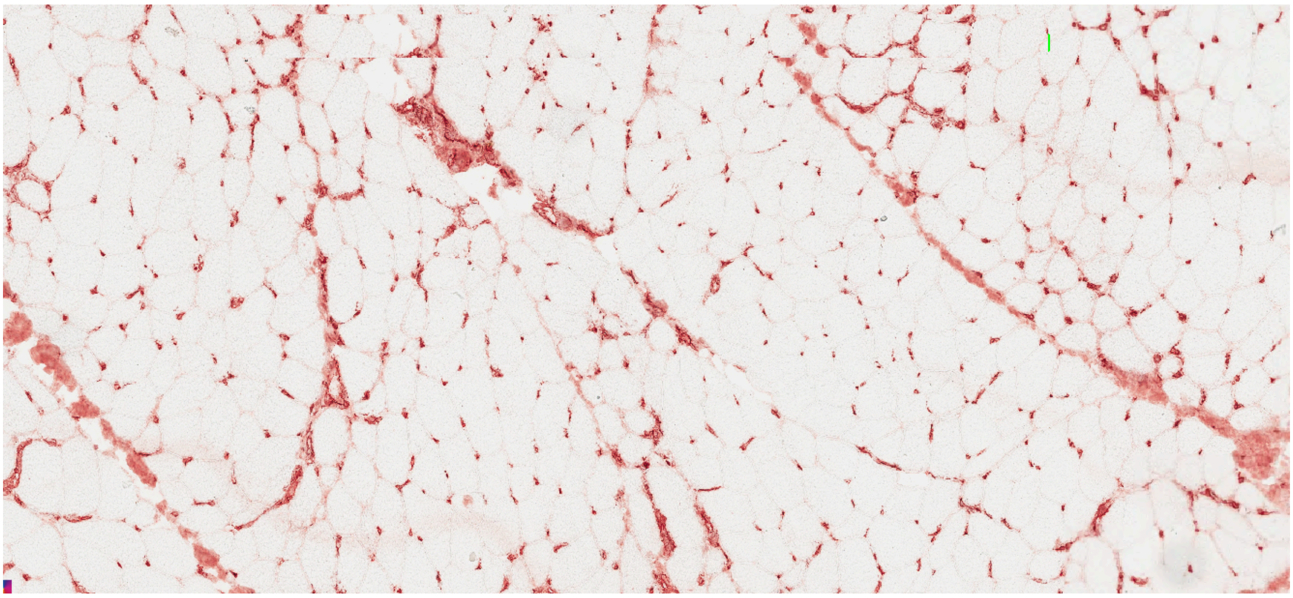

Class 2 extraction

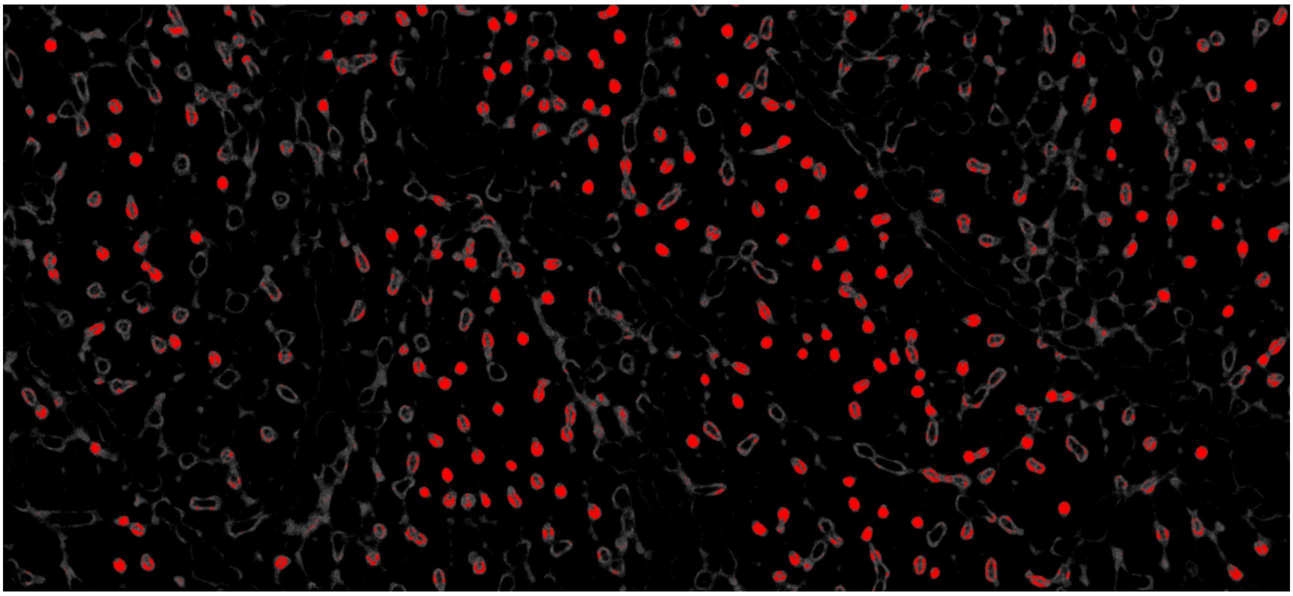

Red Pixel counting (class 2)

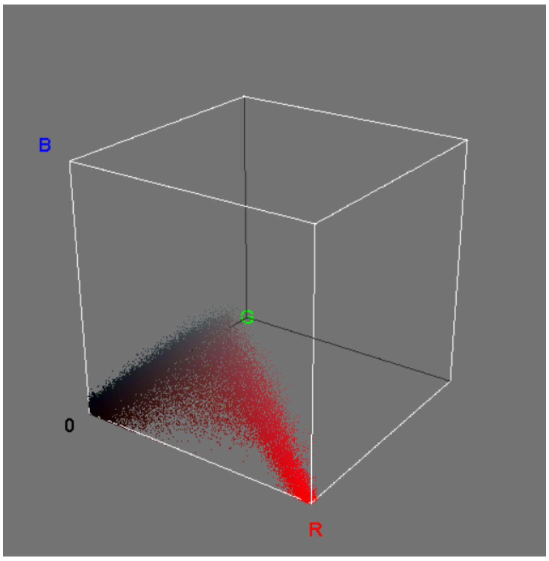

Photo: **1226**

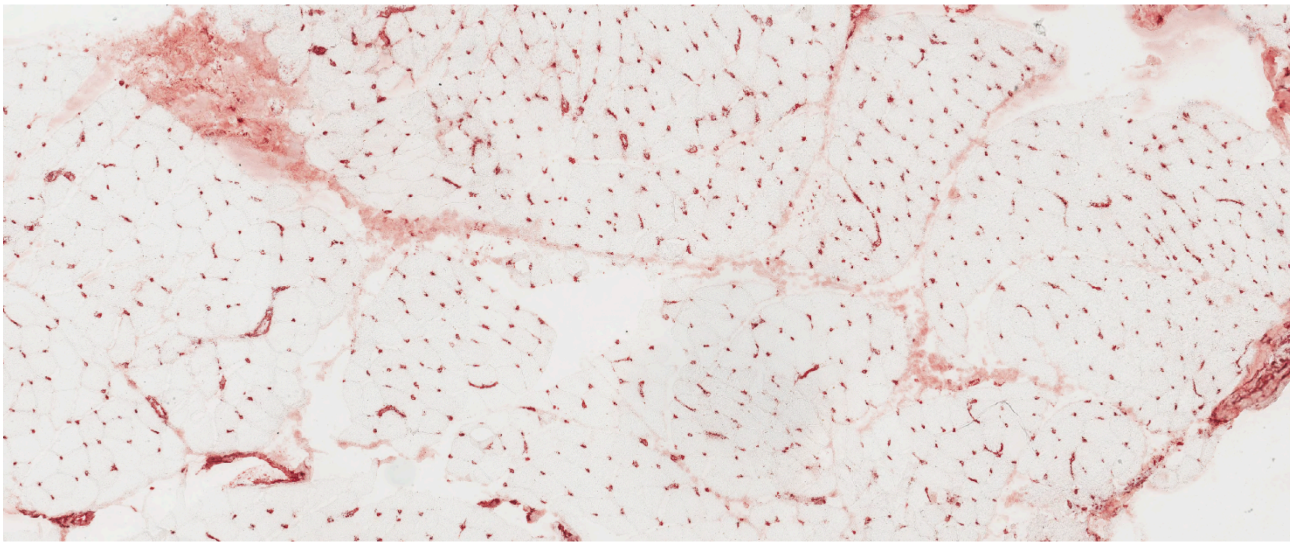

Class 2 extraction

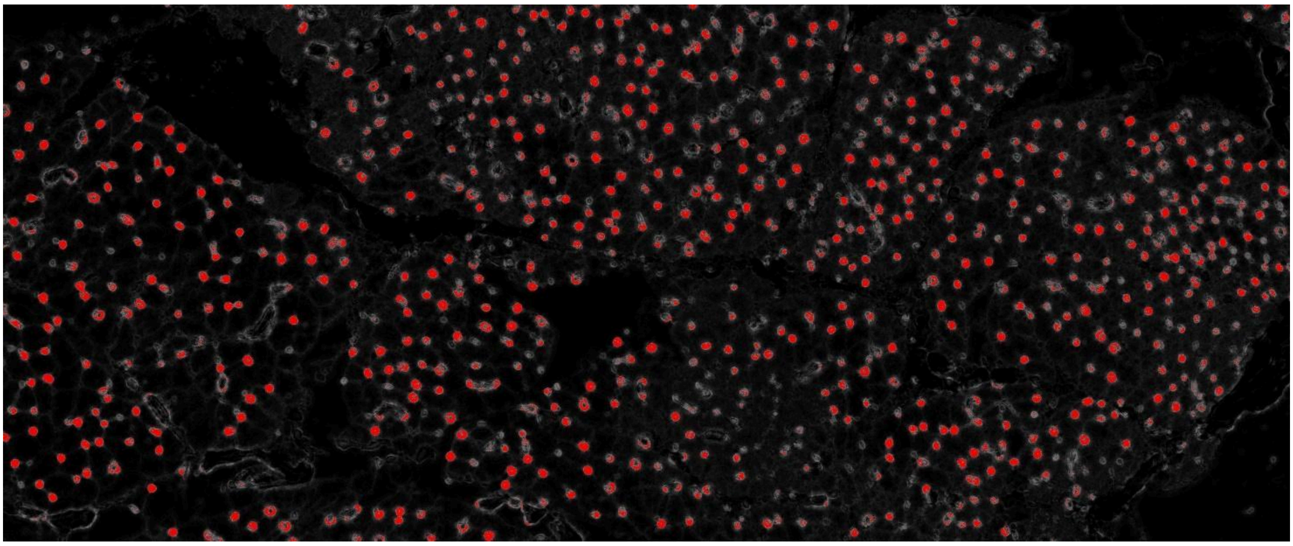

Red Pixel counting (class 2)

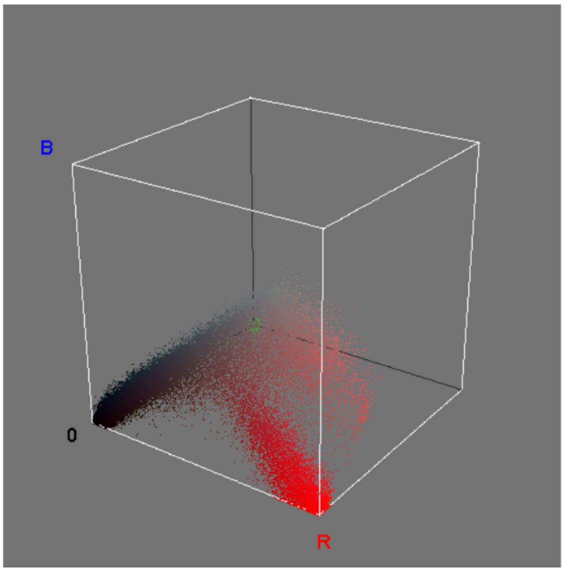

Photo: **1437**

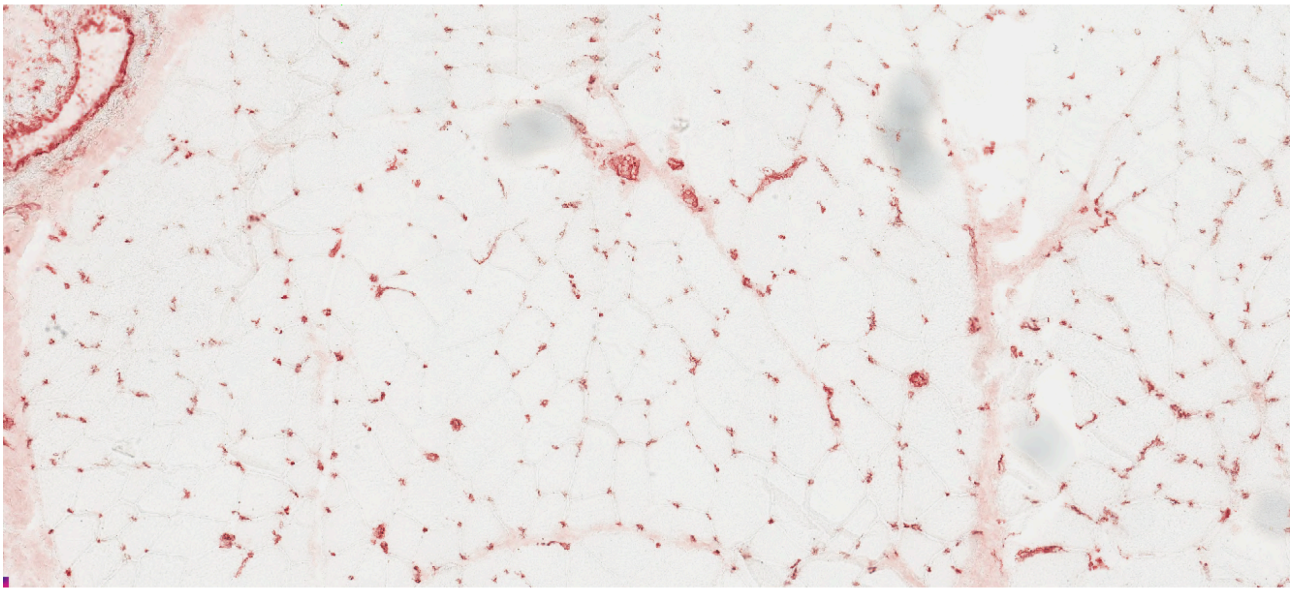

Class 2 extraction

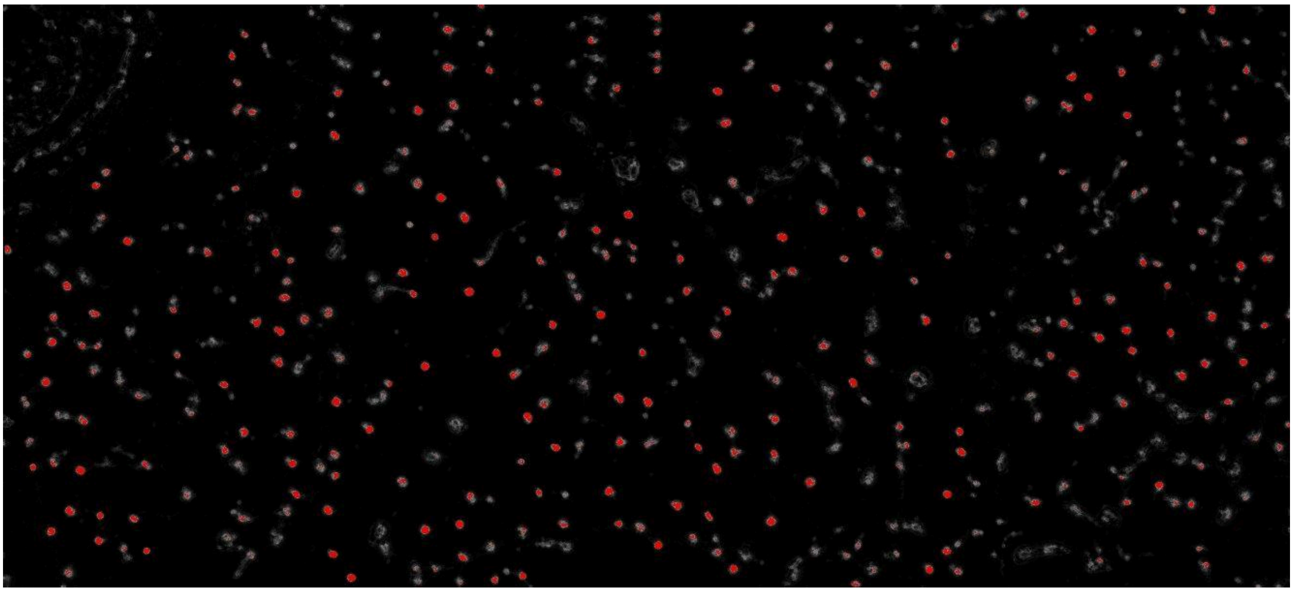

Red Pixel counting (class 2)

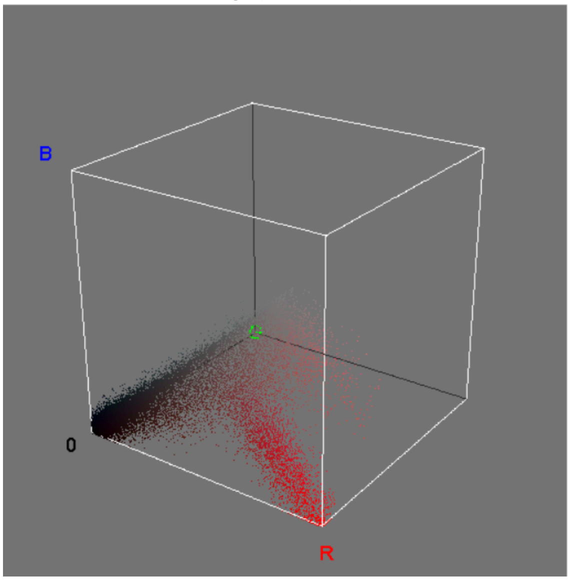

Photo: **1387**

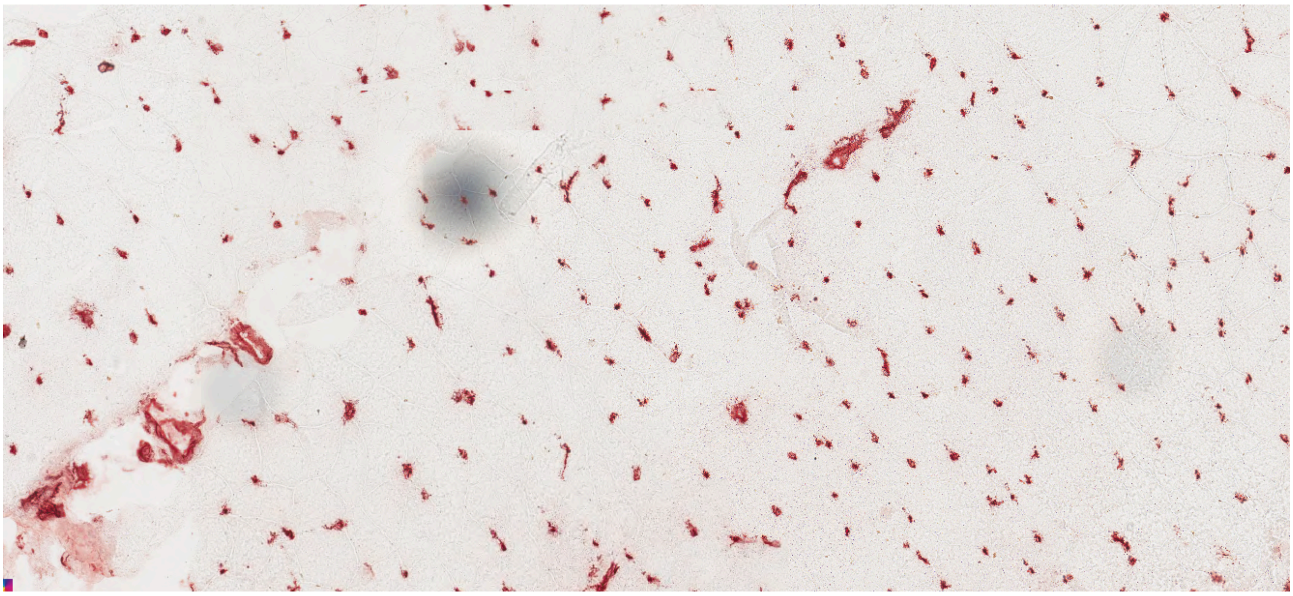

Class 2 extraction

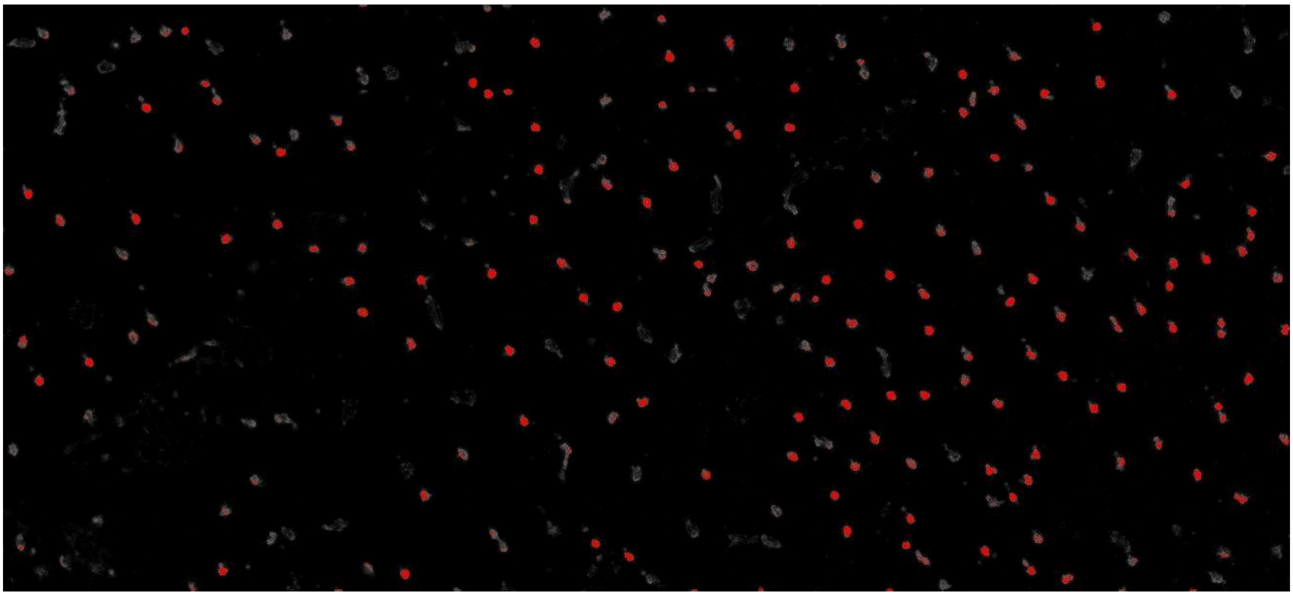

Red Pixel counting (class 2)

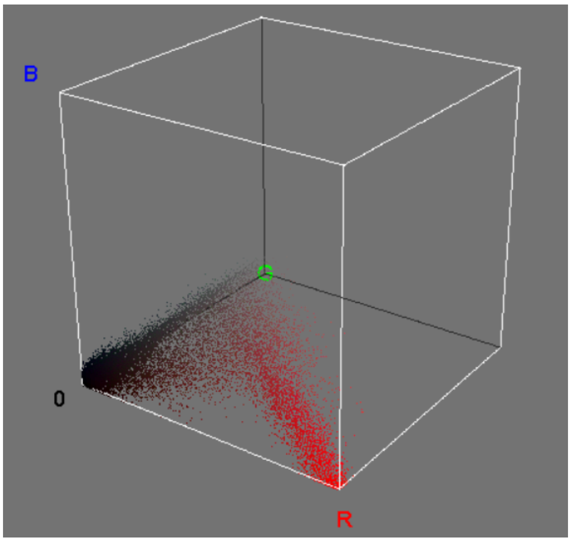

Photo: **1420**

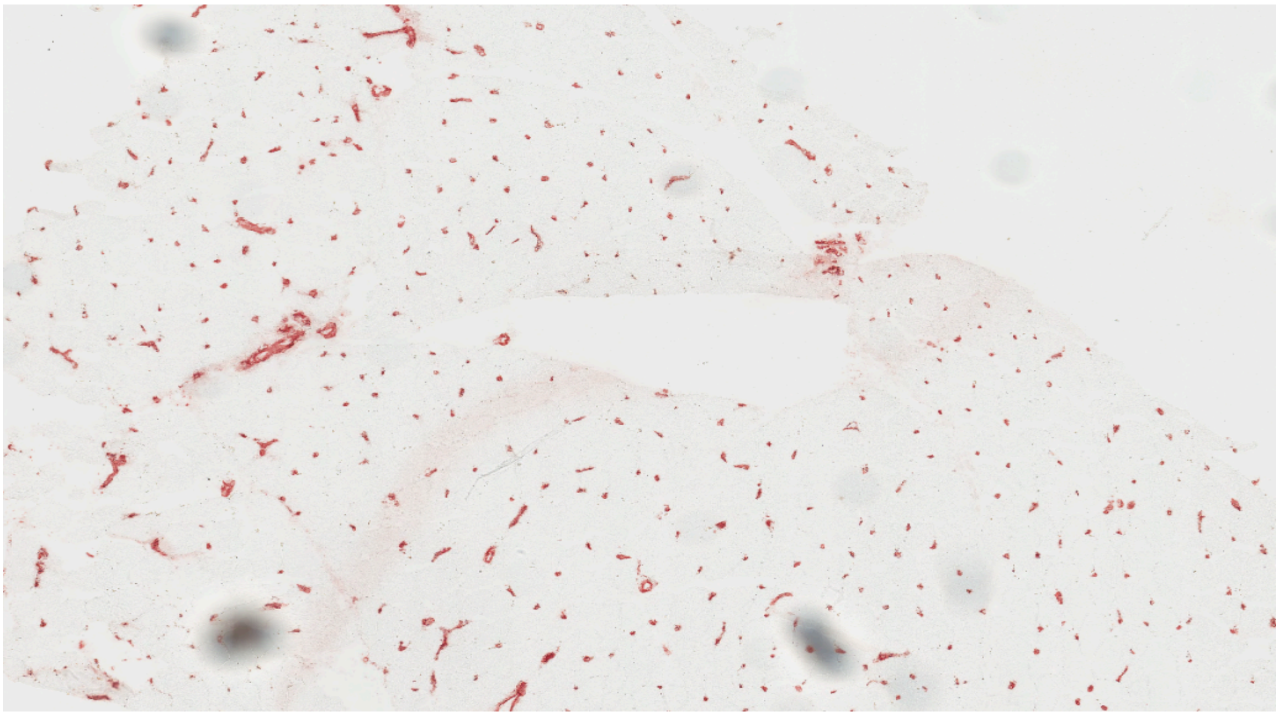

Class 2 extraction

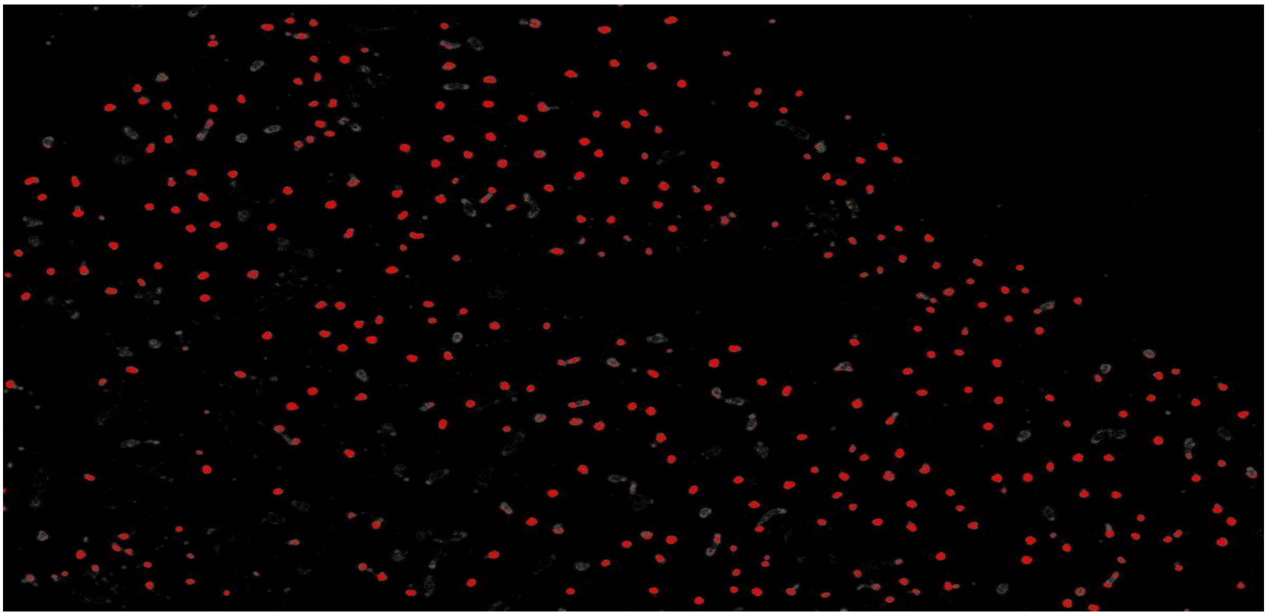

Red Pixel counting (class 2)

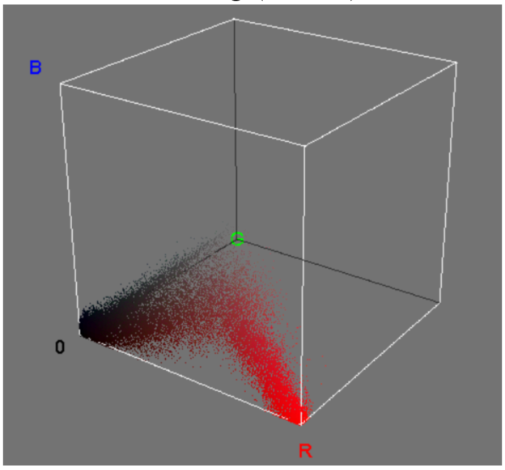

Photo: **1441**

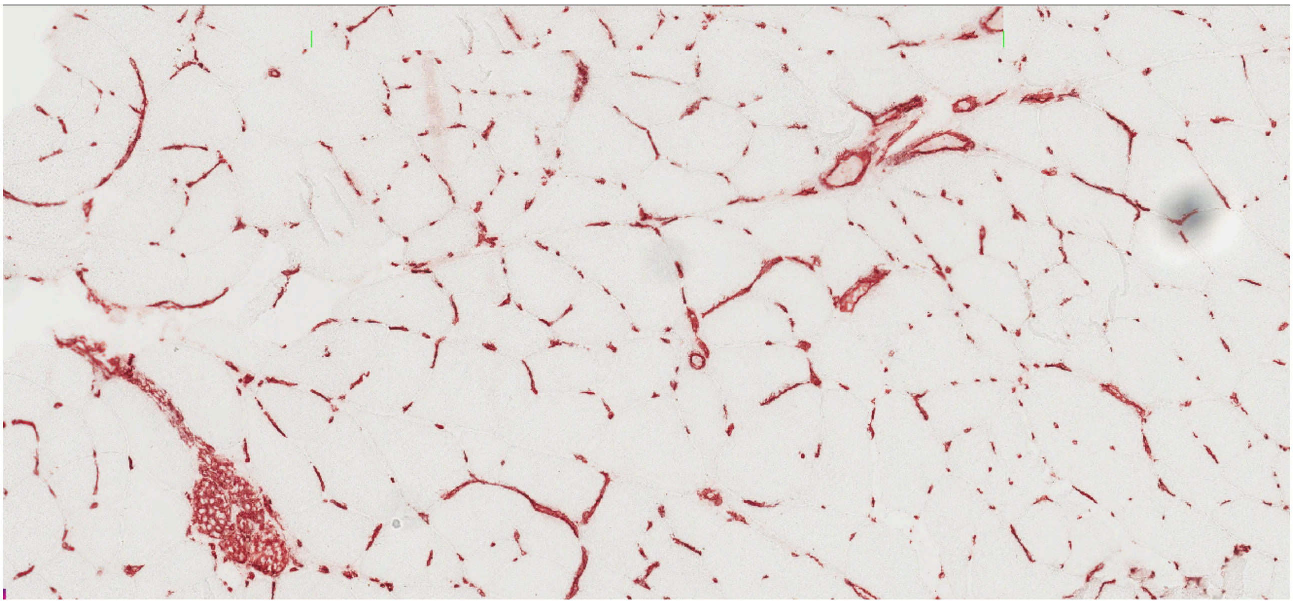

Class 2 extraction

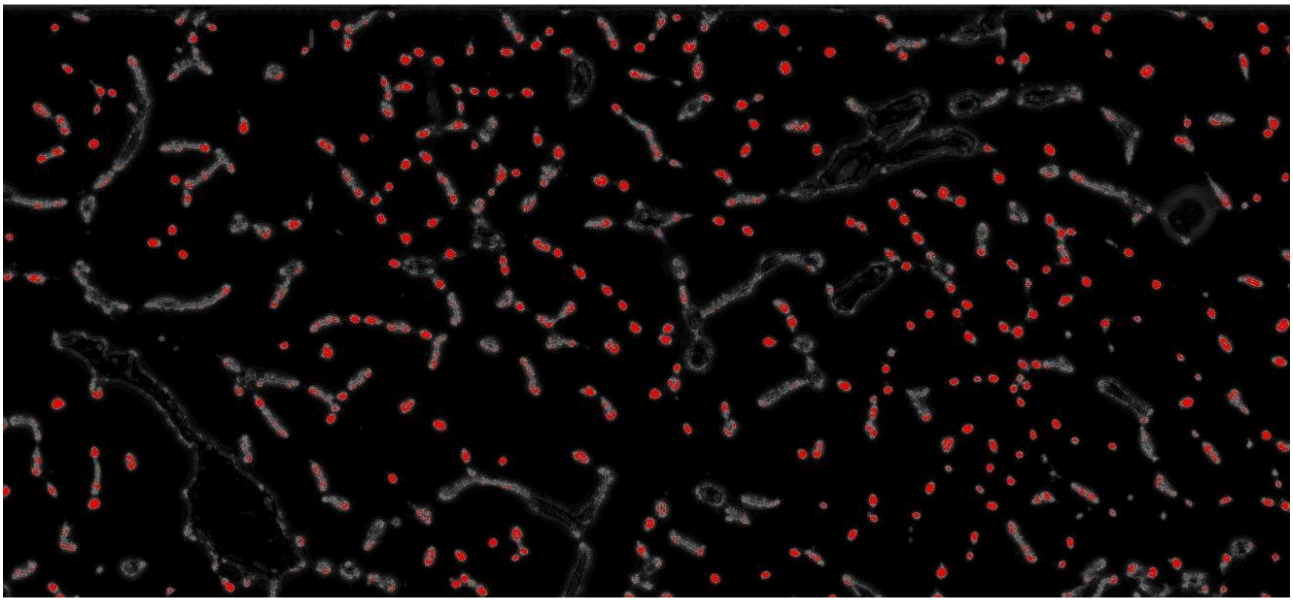

Red Pixel counting (class 2)

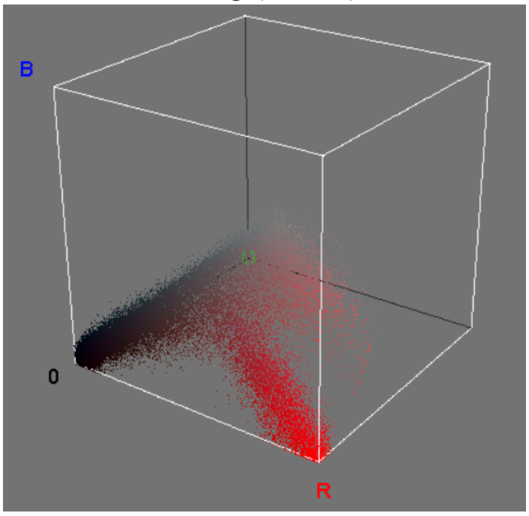

Photo: **1447**

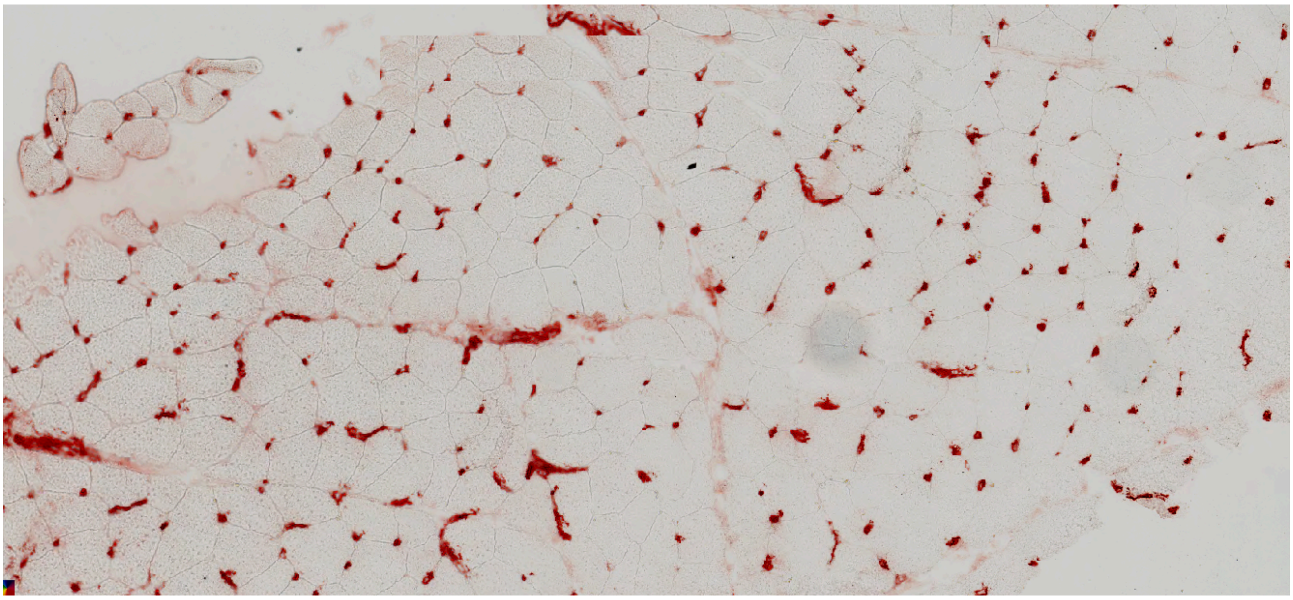

Class 2 extraction

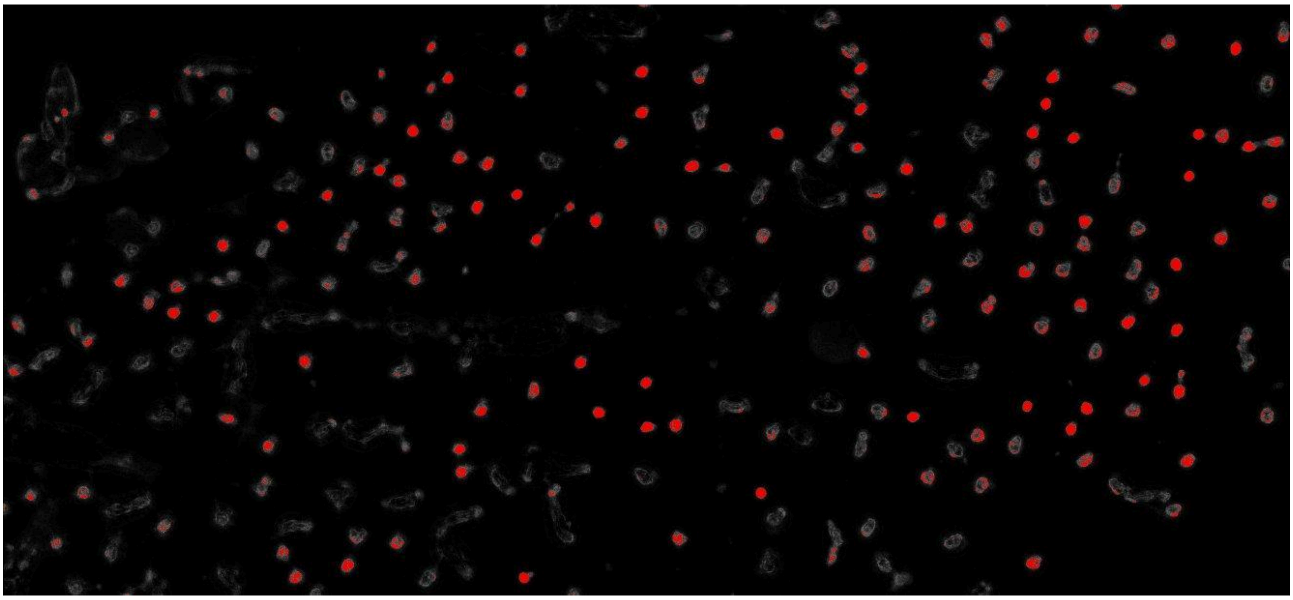

Red Pixel counting (class 2)

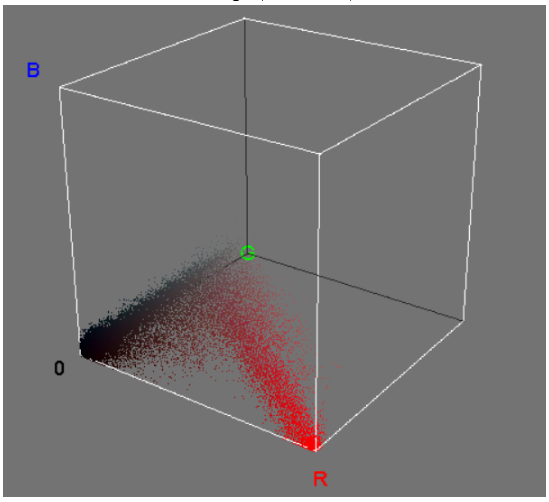

Photo: **1456**

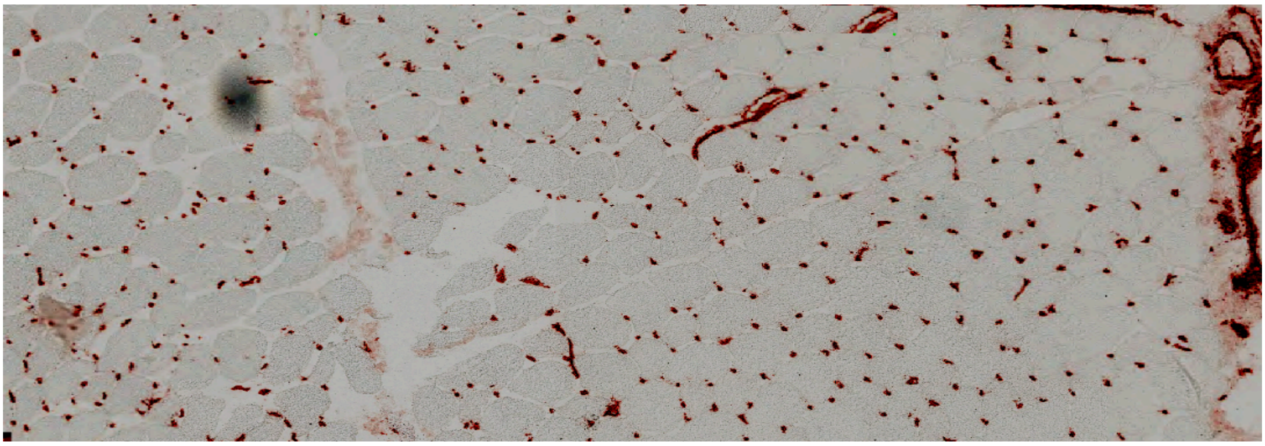

Class 2 extraction

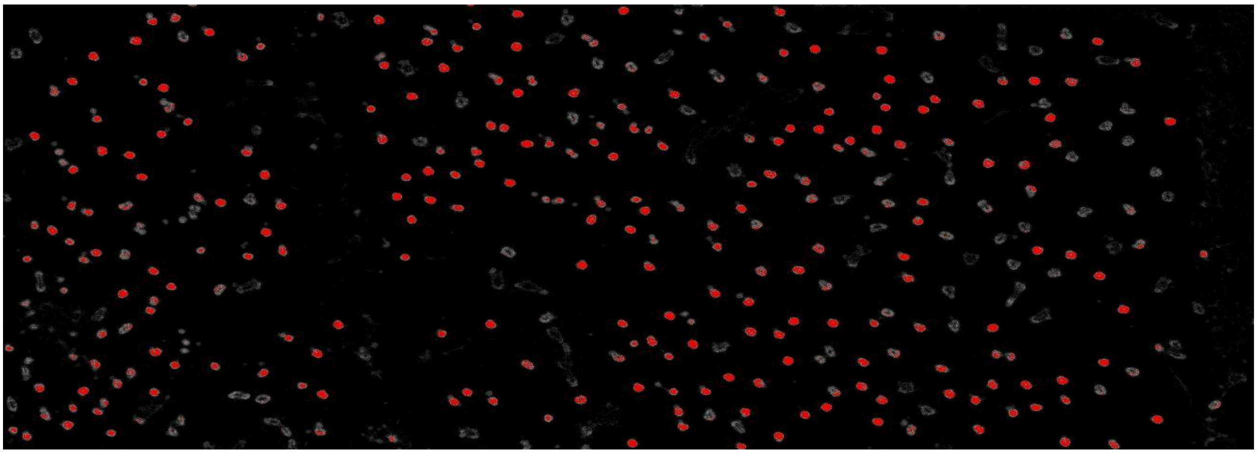

Red Pixel counting (class 2)

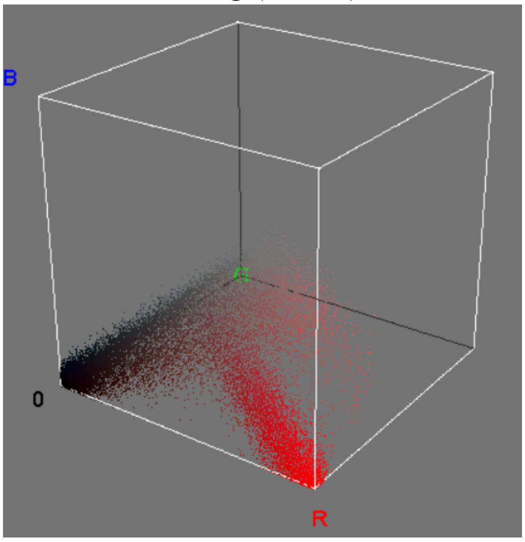

Photo: **1505**

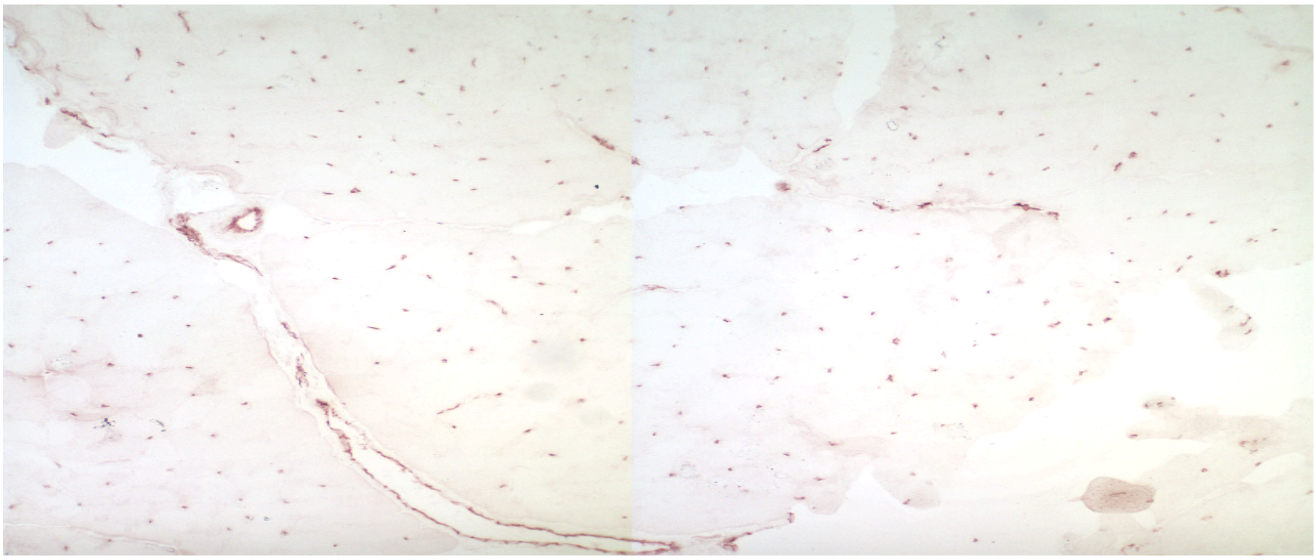

Class 2 extraction

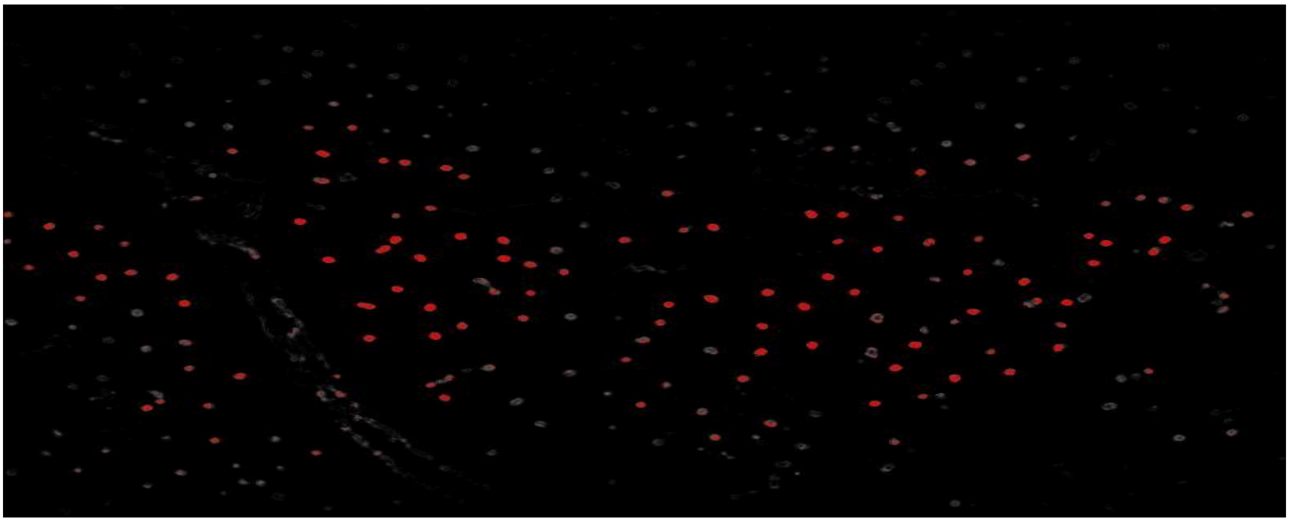

Red Pixel counting (class 2)

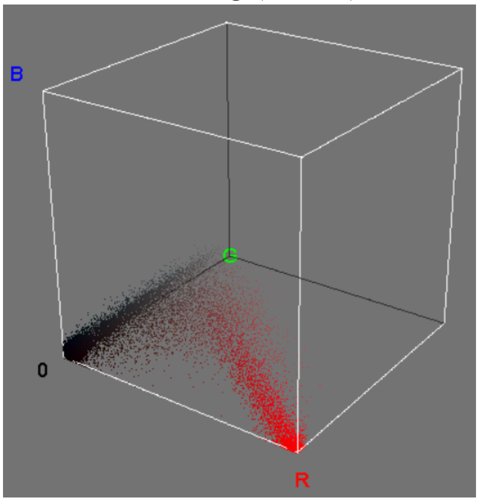

Photo: **1534**

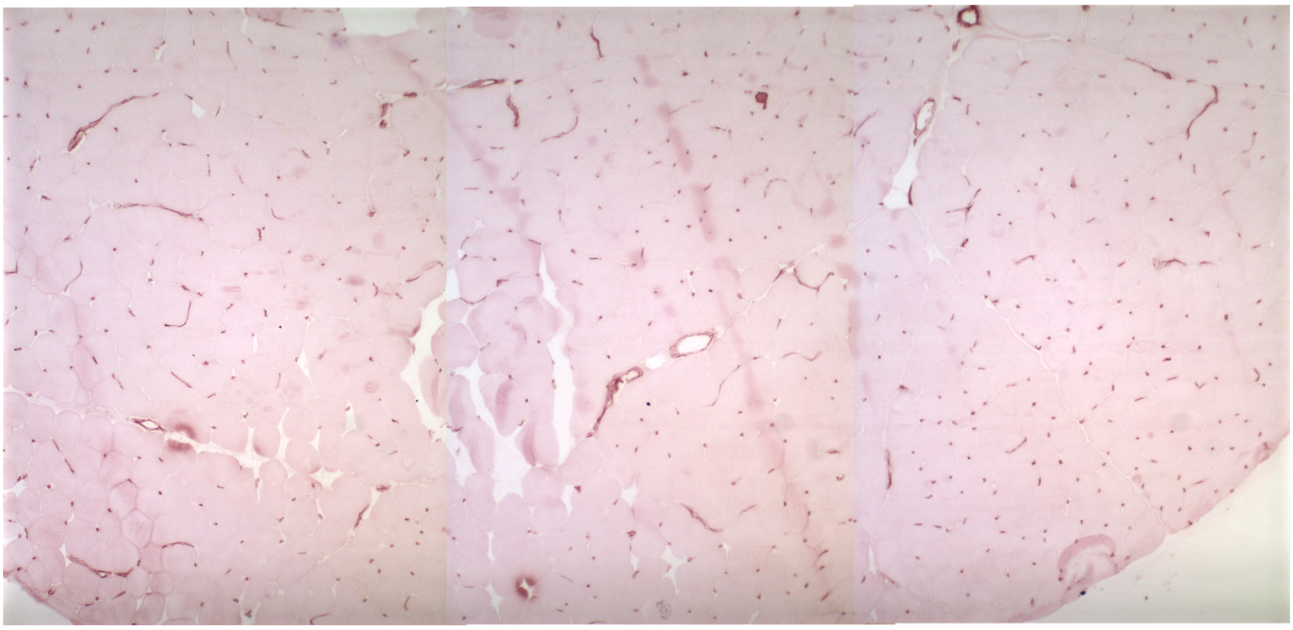

Class 2 extraction

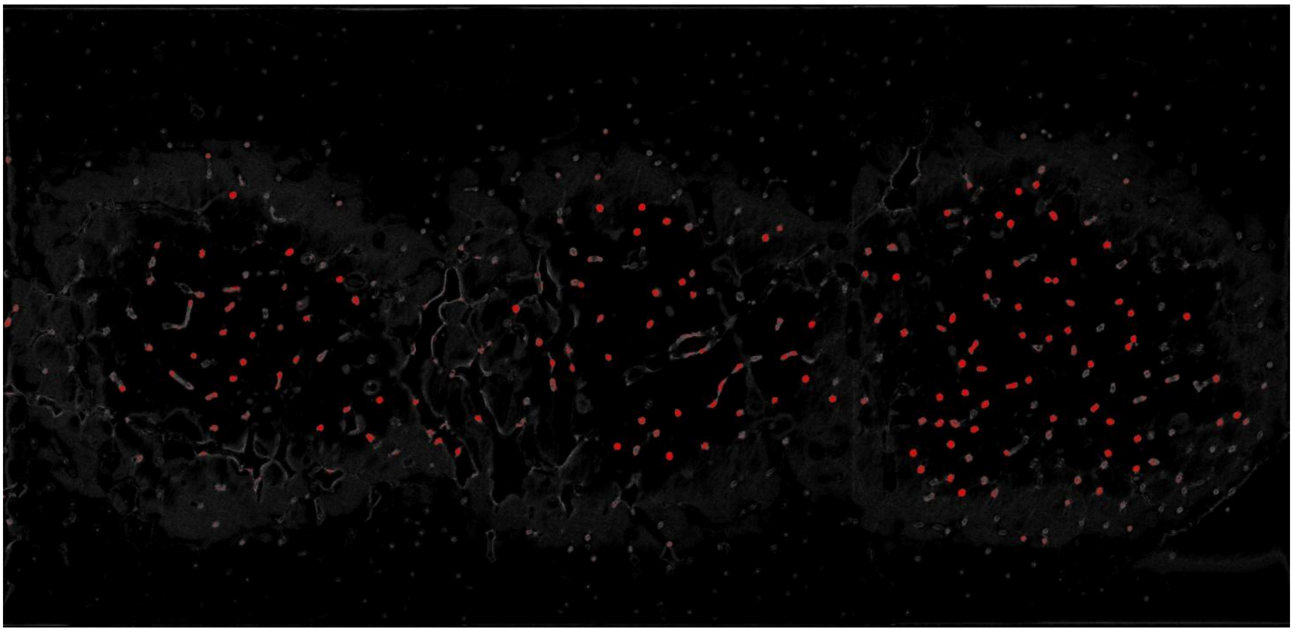

Red Pixel counting (class 2)

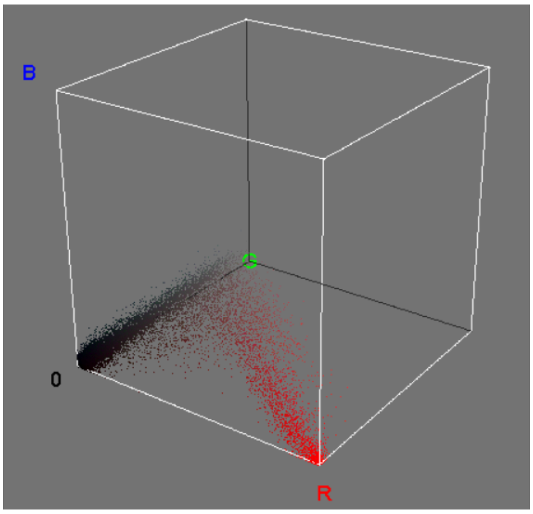

TABLE I
SUMMARY OF FRF TESTING RESULTS

**Photo**	**Total image area [pixel^2]**	**Red pixel percentage (class 2) [%]**
1326	1257377	4,254
1226	2740916	3,733
1364	812520	4,82
1365	818620	5,855
1437	799456	3,121
1387	807917	2,568
1420	969624	3,032
1441	808964	5,57
1447	813241	3,364
1456	809470	4,013
1505	3069087	1,017
1534	5371290	0,879
